# The antimicrobial peptide Magainin-2 interacts with BamA impairing folding of *E. coli* membrane proteins

**DOI:** 10.3389/fchem.2022.1013788

**Published:** 2022-10-17

**Authors:** Angela Di Somma, Carolina Cané, Antonio Moretta, Anna Illiano, Gabriella Pinto, Domenico Cavasso, Angela Amoresano, Luigi Paduano, Angela Duilio

**Affiliations:** ^1^ Department of Chemical Sciences, University of Naples “Federico II”, Naples, Italy; ^2^ National Institute of Biostructures and Biosystems (INBB), Rome, Italy; ^3^ Institut NeuroMyoGène, Unité Physiopathologie et Génétique du Neurone et du Muscle, CNRS UMR5261, INSERM U1315, Université Claude Bernard Lyon 1, Lyon, France

**Keywords:** antimicrobial peptides, proteomics, membrane proteins, mechanism of action, structural and functional characterization

## Abstract

Antimicrobial peptides (AMPs) are a unique and diverse group of molecules endowed with a broad spectrum of antibiotics properties that are being considered as new alternative therapeutic agents. Most of these peptides are membrane-active molecules, killing bacteria by membrane disruption. However, recently an increasing number of AMPs was shown to enter bacterial cells and target intracellular processes fundamental for bacterial life. In this paper we investigated the mechanism of action of Maganin-2 (Mag-2), a well-known antimicrobial peptide isolated from the African clawed frog *Xenopus laevis*, by functional proteomic approaches. Several proteins belonging to *E. coli* macromolecular membrane complexes were identified as Mag-2 putative interactors. Among these, we focused our attention on BamA a membrane protein belonging to the BAM complex responsible for the folding and insertion of nascent β-barrel Outer Membrane Proteins (OMPs) in the outer membrane. *In silico* predictions by molecular modelling, *in vitro* fluorescence binding and Light Scattering experiments carried out using a recombinant form of BamA confirmed the formation of a stable Mag-2/BamA complex and indicated a high affinity of the peptide for BamA. Functional implications of this interactions were investigated by two alternative and complementary approaches. The amount of outer membrane proteins OmpA and OmpF produced in *E. coli* following Mag-2 incubation were evaluated by both western blot analysis and quantitative tandem mass spectrometry in Multiple Reaction Monitoring scan mode. In both experiments a gradual decrease in outer membrane proteins production with time was observed as a consequence of Mag-2 treatment. These results suggested BamA as a possible good target for the rational design of new antibiotics since this protein is responsible for a crucial biological event of bacterial life and is absent in humans.

## 1 Introduction

Antimicrobial peptides (AMPs) are small molecules consisting of 10–100 amino acid residues (Moretta et al., 2020) produced by all organisms. In the last few years, these compounds are being considered as new alternative therapeutic agents for their rapid bactericidal activity, their broad spectrum of action against both Gram-positive and Gram-negative bacteria, fungi and viruses, and their immunomodulatory activity (Amerikova et al., 2019).

AMPs are usually cationic, with a highly positive net charge (+2 to +9), and amphipathic molecules, as their structure includes both hydrophobic and hydrophilic moieties (Torres et al., 2019). They can display powerful antimicrobial activities against antibiotic-resistant bacteria acting at the level of bacterial membranes with several mechanisms, depending on the molecular properties of the peptides themselves and the lipid composition of the membranes (Zhang et al., 2021). Their specific mode of action differs from those of common drugs, thus not allowing the development of drug resistance (Moravej et al., 2018).

Biophysical studies led to hypothesize three models that can explain the membrane disruption by AMPs: *barrel-stave*, *toroidal pore* and *carpet* mechanisms (Di Somma et al., 2020). According to these mechanisms, antimicrobial peptides interact with bacterial outer membranes, perturbing their integrity and causing their disaggregation (Huang, 2006). Most of AMPs show pore formation after binding to the membrane surface. X-ray crystallization studies and spectroscopic analyses reported that Alamethicin, a channel-forming peptide, insert into the lipid bilayer to form a barrel-stave pore structure consisting of eight alamethicin helices (Qian et al., 2008). Melittin, a peptide isolated from bee venom, is a basic amphiphilic peptide, which mainly acts on the lipid matrix of membranes. Fluorescence studies with phospholipid vesicles reported the formation of pore coupled with the translocation of peptide across the lipid bilayer (Lohner and Blondelle, 2005).

Magainins, a group of AMPs derived from the African clawed frog *Xenopus laevis*, exhibit a net positive charge showing greater selectivity for negatively charged sites on the microbial membranes (McMillan and Coombs, 2020). Peptide binding is favoured by both hydrophobic interactions between non-polar amino acids and the hydrophobic nucleus of the membrane and electrostatic interactions between the positive charges of the peptides and the negative charges of lipids (Bahar and Ren, 2013). Although the mechanism of action is not yet fully understood at molecular level, it is known that Magainin-2 (Mag-2) exerts its antimicrobial activity according to a toroidal mechanism (Billah et al., 2022). The peptide induces a high curvature in the bacterial membrane leading to the formation of pores causing membrane dysfunction and the loss of essential contents from the inside of the cell, eventually leading to cell death (Dennison et al., 2014).

However, the molecular events underlying this mechanism are still far from being understood. Investigation of Mag-2 behaviour is mainly focused on the interaction of the peptide with lipid membrane components and the lipopolysaccharide (LPS) molecules (Ding et al., 2003). In this work, we elucidated the mechanism of action of Mag-2 on *Escherichia coli* used as a model with the aims to a) demonstrate that our approach was effective in defining the mechanism of action of this AMP at the molecular level and b) find a possible target to be exploited by antimicrobial compound(s) devoid of dangerous collateral effects. Functional proteomics experiments were exploited for the identification of Mag-2 protein targets identifying several membrane proteins as putative interactors. Among these, we demonstrated a specific interaction of the peptide with BamA, an outer membrane protein belonging to the BAM complex. In Gram-negative bacteria, the BAM complex is responsible for the folding and insertion of nascent β-barrel Outer Membrane Proteins (OMPs) in the outer membrane (Tomasek et al., 2020). The interaction of Mag-2 with BamA was predicted by molecular docking analysis, leading to the identification of peptide-protein interface involved in the binding. This portion of the BamA protein was produced in recombinant form and the peptide-protein interaction was confirmed *in vitro* by fluorescence assay Light Scattering Measurements. Finally, the functional role of Mag-2/BamA interaction in impairing the folding of the membrane protein OmpA was investigated by both western blot experiments and tandem mass spectrometry analyses carried out in Multiple Reaction Monitoring (MRM) scan mode. These approaches demonstrated a clear decrease in *E. coli* OmpA production following incubation with Mag-2. Since Mag-2 has significant toxicity against human cells, these observations can lead to the development of new peptides or peptidomimetic drugs based on the specific interaction with BamA, considered a potential target for novel antibiotics.

## 2 Experimental section

### 2.1 *Escherichia coli* membrane proteins extraction

*E. coli* cells were inoculated in 10 ml of LB liquid medium (Luria-Bertani) and incubated at 37°C for 16 h under stirring. Then, bacterial cells were grown in 1 L at 37°C under stirring for 3 h. The pellet was recovered by centrifugation at 4°C for 15 min at 5,000 rpm, resuspended in 5 ml of Cell Lysis Buffer (20 mM Tris-HCl pH 8.0, 500 mM NaCl, 4 mM DTT, 1 mM PMSF) and subjected to mechanical lysis by sonication. The sample was centrifuged at 4°C for 30 min at 15,000 rpm to pellet unlysed cells and cellular debris and the recovered supernatant was ultracentrifuged for 2 h at 4°C at 54,000 rpm. The obtained pellet was resuspended in 1 ml per gram of pellet of membrane resuspension buffer (50 mM Tris-HCl pH 8.0, 500 mM NaCl, 10% Glycerol, 4 mM DTT, 1 mM PMSF, 6 mM 3 [(3-Cholamidopropyl) dimethylammonium]-1-propanesulfonate (CHAPS) under stirring at 4°C for 16 h. The sample was ultracentrifuged at 4°C for 2 h at 54,000 rpm. The supernatants containing the solubilized membrane proteins were collected (Newby et al., 2009).

### 2.2 Pull down experiment

The pull-down experiment was performed using 200 μL of dried avidin-conjugated agarose beads. A resin with free agarose beads was used for the pre-cleaning, and a resin incubated with a solution of 2 mg/ml of biotinylated Mag-2 for 30 min at 4°C under stirring was used for the pull-down assay. The supernatant was removed by centrifugation at 4°C for 10 min at 3,000 rpm and the resin equilibrated with five volumes of binding buffer at 4 °C. On the pre-cleaning resin, 2.5 mg of membrane proteins were incubated at 4°C for 2 h under stirring to remove possible non-specific interactors. Indeed, only the supernatant containing the unbound membrane proteins was recovered by centrifugation at 4°C for 10 min at 3,000 rpm and then transferred on the pull-down resin for 3 h at 4°C under stirring. The beads were washed with five volumes of binding buffer and the peptide-interacting proteins were released by competitive elution with 500 µL of elution buffer containing an excess of biotin (2 mM) for 1 h at 4°C under stirring. Mag-2 putative protein interactors were fractionated by SDS-PAGE and the protein bands from both the sample and the control lanes were excised from the gel and subjected to *in situ* hydrolysis with trypsin. The resulting peptide mixtures were directly analyzed by Liquid Chromatography/Tandem Mass Spectrometry (LC-MS/MS) using an LTQ Orbitrap XLOrbitrap mass spectrometer (Thermo Fisher Scientific, Bremen, Germany) and the obtained data were used to search for a non-redundant protein database using an inhouse version of the Mascot software, leading to the identification of the putative AMP protein interactors. Proteins identified both in the control and in the sample were discarded, whereas those occurring in the sample and absent in the control were considered as putative Mag-2 interactors. The putative peptide interactors were gathered within functional pathways by the bioinformatic tools DAVID (Huang da et al., 2009), KEGG (Kanehisa et al., 2017), and STRING (Szklarczyk et al., 2021).

### 2.3 Mag-2/BamA molecular docking analysis

The putative binding sites of Mag-2 peptide on BamA protein were determined through molecular docking calculations. Both peptide and protein have been modelled using I-TASSER Server (Roy et al., 2010; Yang et al., 2015; Yang and Zhang, 2015). The Magainin-2-BamA complex model has been obtained using PatchDock Server (Schneidman-Duhovny et al., 2005) and the structures have been refined with FireDock Server (Mashiach et al., 2008), which also gives the Global Energy, the Attractive and Repulsive Van der Waals (VdW) forces and the Atomic Contact Energy (ACE) values of the complex. All the interactions and the amino acids involved at the interface were determined using the PDBsum Server (Laskowski, 2001; Laskowski, 2009; De Beer et al., 2014). The Gibbs free energy, ΔG, and the dissociation constant, Kd, of the protein-peptide complex have been predicted using the PRODIGY webserver (Xue et al., 2016). All the figures have been generated through UCSF CHIMERA software (Pettersen et al., 2004).

### 2.4 Expression of *Escherichia coli* BamA_p5_

6XHis-tagged *E. coli* BamAp5 consisting of the β-barrel domain and the fifth POTRA domain (residues 344–810) was expressed in the pet30b_BamA_PD5 plasmid in *E. coli* BL21 cells and then cells were used to inoculate 1L of LB culture media with 50 μg/ml kanamycin. The culture was incubated at 37 °C until the ODλ = 600 reached 0.5 OD/mL value and then protein expression was induced by adding Isopropyl β-d-1-thiogalactopyranoside (IPTG) to a final concentration of 1 mM. The culture was incubated at 37°C for 3 h. Cells were then harvested by centrifugation for 15 min at 4°C at 5,000 rpm and the obtained pellets were resuspended in lysis buffer (150 mM NaCl, 20 mM Tris pH 8.0, 1 mg/ml lysozyme). Afterwards cells were lysed on ice by sonication and the suspension were centrifugated for 20 min at 4°C. The pellet containing the protein was washed with lysis buffer and resuspended in lysis buffer containing 5 mg/ml lysozyme and 1% triton. The solution was stirred for 3 h and centrifugated for 20 min at 4°C at 15,000 rpm. This step was repeated in the absence of lysozyme followed by another centrifugation step. The pellet was washed with lysis buffer to remove the detergent and centrifuged for 20 min at 4°C at 15,000 rpm. The obtained pellet was resuspended in denaturing buffer (6 M Gu–HCl, 20 mM Tris pH 8.5), homogenized using ultraturax and centrifugated.

### 2.5 BamA_p5_ purification and primary structure characterization

The BamA_p5_ protein was purified by affinity chromatography, using a Ni-NTA column equilibrated with five column volumes of denaturing buffer, followed by five column volumes of the same buffer containing 20 mM imidazole to remove unbound material. BamA_P5_ was eluted with denaturing buffer containing 300 mM imidazole. The eluted protein was refolded by adding dropwise to ten times the volume of Tris 20 mM pH 8.4 and Triton 0.5% for 12 h at 8°C under stirring. The solution was diluted with 20 mM Tris pH 8.4 until 0.2% triton concentration was reached (Albrecht et al., 2014). Protein concentration was estimated with Bradford assay (Bio-Rad protein assay) and protein purity was assessed by Sodium Dodecyl Sulfate-polyacrylamide gel electrophoresis (SDS-PAGE). Primary structure of BamA was validate by mass mapping using MALDI-TOF and circular dichroism (CD) analysis was performed to verify the correct folding of the protein, using a JASCO J-715 spectropolarimeter equipped with a Peltier thermostatic cell holder (Model PTC-348WI) in 1 cm optical path-length quartz cell. CD spectra were acquired in the range 190–250 nm, performing three accumulations for each measure, a scanning speed of 100 nm min^−1^ and data pitch of 0.2 nm.

### 2.6 Binding experiment

Fluorescence experiments were performed on a Fluoromax-4 spectrofluorometer from Horiba Scientific, using 1 cm optical path-length quartz cell under controlled temperature conditions (Peltier control system at 20°C). Titrations were carried out in 20 mM Tris-HCl pH 8.4, 0.2% triton. The intrinsic fluorescence intensity of BamA_P5_ at a concentration of 3.7 × 10^−6^ M was monitored at 280 nm (slit 4 nm) and the emission was monitored at 308 nm (slit 4 nm) without and in the presence of increasing concentrations of Mag-2 peptide (from 2 to 18.64 µM). All experiments were performed in duplicate. The change in the fluorescence intensity of the reaction set was fit into “one site-specific binding” equation of GraphPad Prism 5 (GraphPad Software).

### 2.7 Dynamic light scattering

DLS measurements were performed with a homemade instrument composed by a Photocor compact goniometer, an SMD 6000 Laser Quantum 50 mW light source operating at 532.5 nm, a photomultiplier (PMT-120-OP/B), and a correlator (Flex 02-01 D) from Correlator.com. The temperature was controlled with a thermostat bath.

In DLS, the intensity autocorrelation function g^(2) (^t) is measured and related to the electric field autocorrelation function g^(1) (^t) by the Siegert relation. This latter function can be written as the inverse Laplace transform of the distribution of the relaxation rate *Γ* used to calculate the translational diffusion coefficient D = Γ/q^2^:

$$g^{\left(1\right)}\left(t\right)=\overset{+\infty }{\underset{-\infty }{\int }}\tau A\left(\tau \right)\exp\left(-\frac{t}{\tau }\right)d\ln\tau$$ (1)

where τ=1/Γ and q is the modulus of the scattering vector q=4πn/λsin(θ/2) , n_0_ is the refractive index of the solution, λ is the incident wavelength, and θ represents the scattering angle. Laplace transforms were performed using a variation of CONTIN. The measurements were analyzed with “Precision Deconvolve” a program based on the approach of Benedek and Lomakin (Braginskaya et al., 1983). For each sample at least 10 independent measurements were made and through a final assessment by the “regularization” procedure a mean diffusion coefficient was determined.

Diffusion coefficients were then employed to calculate hydrodynamic radii by means of Stokes-Einstein relation:

$$R_{H}=\frac{kT}{6\pi \eta \langle D_{\infty }\rangle }$$ (2)

where *k* is the Boltzmann constant, *T* is the absolute temperature and *η* is the medium viscosity, whose mean value was assumed to be 0.89 cP for each aqueous mixture.

Due to the high dilution, it is possible to make the approximation: $\langle D\rangle \equiv \langle D_{\infty }\rangle$ and $\equiv \eta_{\infty }$ *,* where η represents the solution viscosity. In this hypothesis, Eq. 2 can be reasonably used to estimate the hydrodynamic radius of the aggregates.

### 2.8 Mass spectrometry investigation

#### 2.8.1 In solution digestion

100 μg of *E. coli* membrane proteins were used to perform mass spectrometry analysis. Protein hydrolysis was carried out by using an in-solution digestion protocol reported by Illiano et all. (Illiano et al., 2021). followed by desalting step performed on Pierce C18 spin column (Thermo Scientific Pierce, Waltham, Massachusetts, United States). Desalted peptide mixture was kept at -20 °C until the mass spectrometry analysis.

#### 2.8.2 LC-MRM/MS analysis

The identification of peptides performed by full scan LC-MS/MS analysis was useful to select the best peptides of OmpA and OmpF to be monitored by LC-MS/MS in Multiple Reaction Monitoring (MRM) ion mode.

Skyline software (3.7, 64-bit version MacCoss Lab Software, University of Washington, United States) was used for the selection of the best precursor ion-product ion transitions and instrumental parameters like collision energy (CE), dwell time and cone voltage. Peptides with zero missed cleavages were considered and the best two to five transitions per peptide were selected from the top ranked y- and b-fragments. As a result, 18 peptides for OmpA and OmpF target proteins were selected, and 140 transitions were monitored during a single analysis.

Table 1 summarizes the amino acid sequence for the selected peptides, the m/z of precursor ion and product ions and the corresponding Collision energy (CE) values to set up the MRM-MS method.

**TABLE 1:** *E. coli* proteins identified as putative Mag-2 interactors.

Proteins	Peptides	SwissProt Code
Protein OmpA	13	P0A910
Protein OmpN	2	P77747
Protein OmpC	2	P06996
Protein OmpF	6	P02931
Porin NmpC	5	P21420
Porin PhoE	5	P02932
Prolipoprotein Lpp	2	P69776
Protein YhcB	4	P0ADW3
Protein TolC	6	P02930
Protein TolB	26	P0A855
Cell division coordinator CpoB	1	P45955
Maltoporin lamB	20	P02943
Multidrug efflux pump AcrA	12	P0AE06
Protein assembly factor BamA	5	P0A940
Protein assembly factor BamB	6	P77774
Protein assembly factor BamC	4	P0A903
Protein assembly factor BamD	7	P0AC02
Dehydratase FabZ	4	P0A6Q6
Modulator of FtsH protease HflK	10	P0ABC7
SecA	5	P0AFY8
FtsJ	1	P0C0R7
Cell division protein FtsZ	20	P0A9A6
Cell division protein FtsA	3	P0ABH0
Rod shape-determ P MreB (MreB)	6	P0A9X4
MurG	4	P17443
MinD	9	P0AEZ3
Elongation factor Tu 1 (TufA)	32	P0CE48
Murein hydrolase activator NlpD	2	P0ADA3
LPS-Assembly protein LptD	2	P31554
Protein DnaA	4	P03004
Protein DnaJ	28	P08622
Transcrip termination Fact Rho (Rho)	5	P0AG30
Bifunctional protein PutA	4	P09546
Protein RecA	14	P0A7G6
LacI	4	P03023

Peptide mixtures were analyzed by LC-MS/MS in MRM ion mode using a Xevo TQ-S (Waters, Milford, MA, United States) equipped with an IonKey UPLC Microflow Source coupled to an UPLC Acquity System (Waters, Milford, MA, United States). For each run, 1 μL of peptide mixture was injected and separated on a TS3 1.0 mm × 150 mm analytical RP column (Waters, Milford, MA, United States) at 45°C with a flow rate of 3 μL min-1 using 0.1% HCOOH in water (LC-MS grade) as eluent A and 0.1% HCOOH in ACN as eluent B. Peptides were eluted (starting from 1 min after injection) with a linear gradient of eluent B in A from 7% to 95% in 55 min. The column was re-equilibrated at initial conditions for 4 min. The MRM mass spectrometric analyses were performed in positive ion mode. The duty cycle was set to automatic and dwell times were minimal 5 ms. Cone voltage was set to 35V.

## 3 Results

### 3.1 Mechanism of action of Mag-2

The mechanism of action of Mag-2 was investigated at the molecular level by functional proteomic experiments. A list of 35 putative protein interactors were identified and listed in Table 1.

**FIGURE 1:**
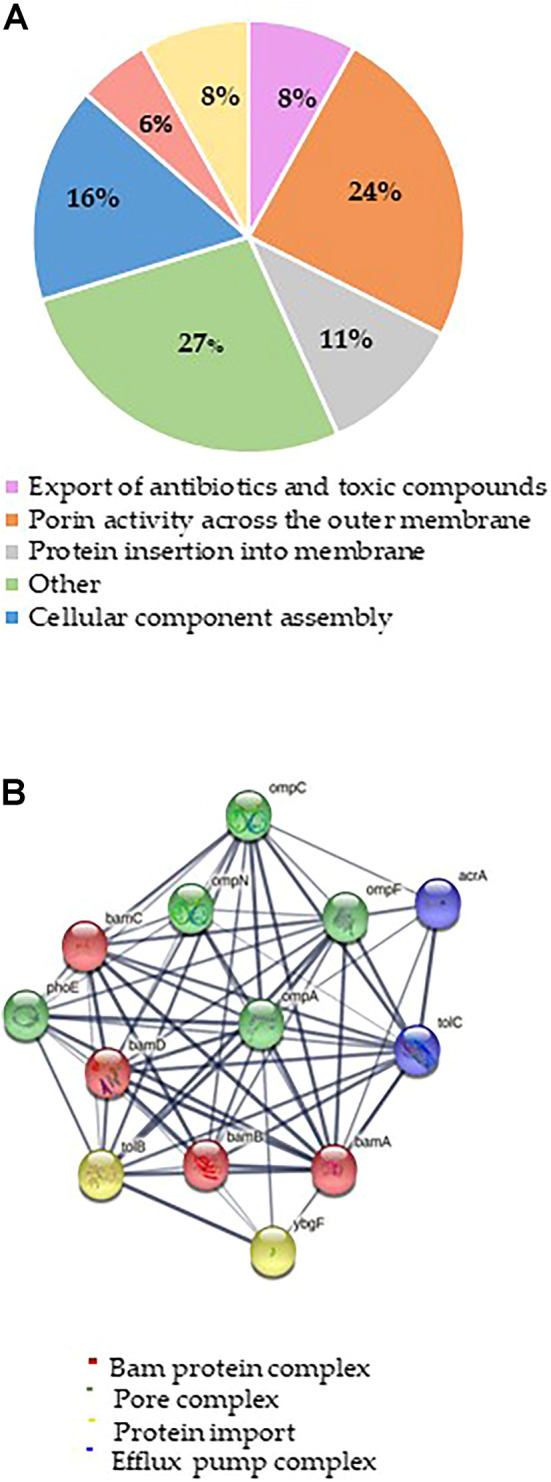
**(A)** Distribution of Mag-2 putative protein partners identified in the pull-down experiment according to their biological functions. **(B)** STRING analysis of the putative Mag-2 interactors belonging to the Bam complex, pore complex, protein import and efflux pump complex showing the occurrence of a network including 13 proteins.

The putative Mag-2 interactors were then grouped according to the biological processes they are involved into as shown in Figure 1A. A bioinformatic analysis using the STRING software was performed and the results are shown in Figure 1B. A large number of protein interactors including Omp N/C/A and the BAM complex (subunits A, B, C and D) gathered within protein complexes involved in porin activity and protein insertion in membrane.

These results suggested a specific interaction of Mag-2 with membrane proteins involved in pore formation supporting the proposed toroidal mechanism. This interaction might then contribute to stabilize transient pores that expand through the membrane allowing the translocation of the peptide into the inner membrane, leading to membrane disruption (Avci et al., 2018).

Among the putative Mag-2 interactors, we focused our attention on the Bam complex as the proteomic experiment led to the identification of all the protein components of this complex essential for bacterial surviving. In particular, BamA is the central component of the Bam complex while BamB, -C, -D and -E are four lipoproteins with a secondary role. BamA is highly conserved in all Gram-negative bacteria and was identified as a target of a small molecules called MRL-494 and IMB-H4 that inhibits the assembly of OMPs in Gram-negative bacteria (https://doi.wrg/10.1073/pnas.1912345116 and 35] and (Hart et al., 2019; Li et al., 2020). According to these considerations, a preliminary molecular docking analysis of the putative BamA-Mag2 complex was carried out.

### 3.2 Molecular docking analysis

**FIGURE 2:**
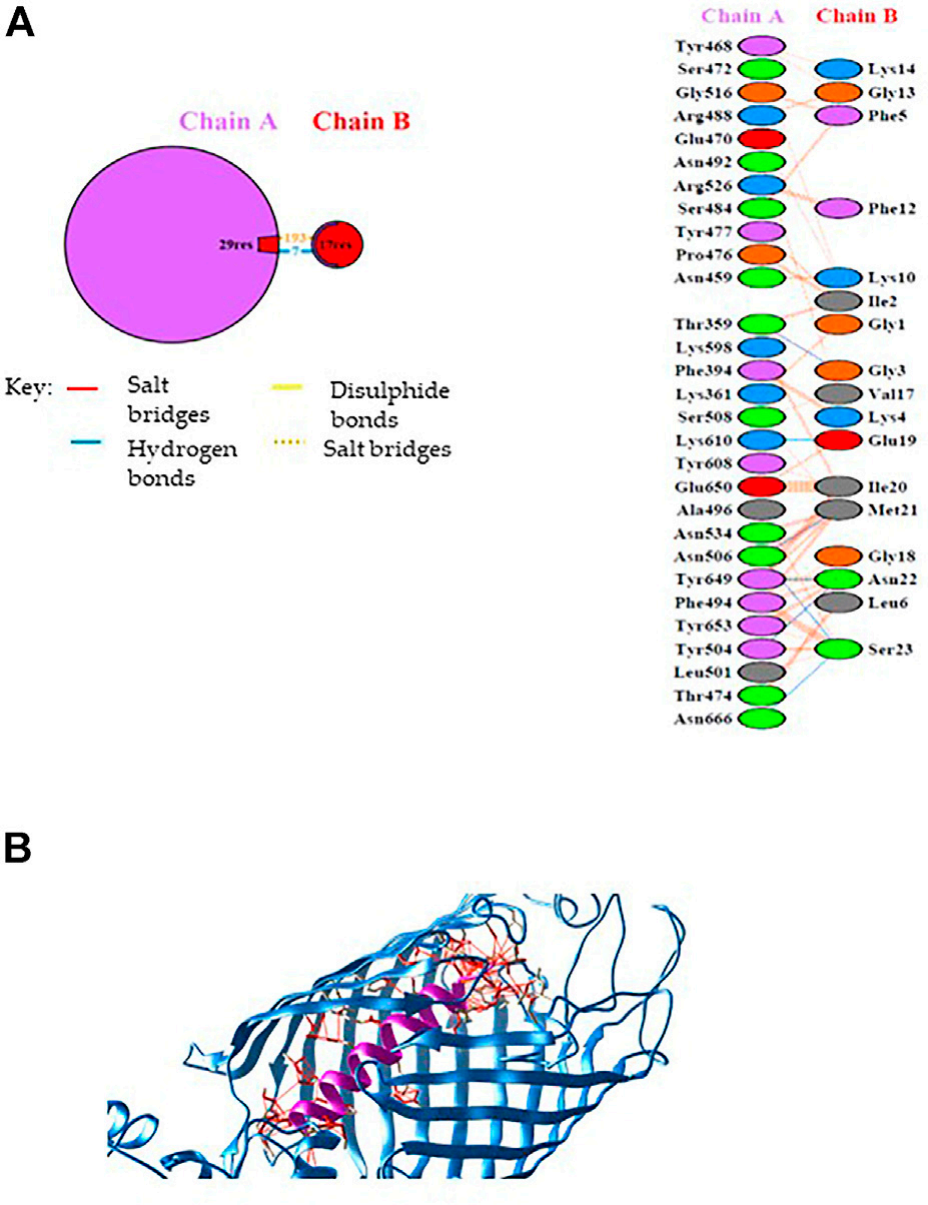
**(A)** Schematic representation of the identified interactions occurring at the protein-peptide interace. **(B)** Protein-peptide model obtained by docking calculations. The BamA protein is in blue while the Magainin-2 peptide is in purple. Interactions at the protein-peptide interface are shown by red lines. The image has been generated with UCSF CHIMERA software (De Beer et al., 2014).

According to the pull-down experiment, BamA, the core component of the outer membrane protein assembly complex (BAM), was identified as a putative Mag-2 interactor. This protein is involved in assembly and insertion of beta-barrel proteins into the outer membrane and acts as a receptor for the contact-dependent growth inhibition (CDI) effector (Aoki et al., 2008). We were then stimulated to confirm this interaction by molecular docking analysis. The Mag-2 peptide and the BamA protein were modelled with the I-TASSER Server (Figure 2), obtaining the best model with a C-score value of -0.57, an estimated TM-score of 0.64 ± 0.13 and an estimated RMSD score value of 2.3 ± 1.8 Å for Mag-2 and a C-score value of 0.76, an estimated TM-score of 0.82 ± 0.09 and an estimated RMSD score value of 6.7 ± 4.0 Å for BamA protein. The docked protein-peptide model, BamA protein (Chain A)—Mag-2 peptide (Chain B), was obtained using the PatchDock Server and the structures have been refined through the FireDock Server, obtaining a Global Energy = -31.57 Kcal/mol; Attractive VdW forces = -36.78 KJ/mol; Repulsive VdW forces = 34.60 KJ/mol and ACE = -3.62 Kcal/mol.

Calculations revealed that Mag-2 may bind the interior cavity of the BamA transmembrane β-barrel domain consisting of 16 antiparallel β strands and involved in the binding of Outer Membrane Proteins (OMP) substrate. A detailed analysis of the interactions at the protein-peptide interface suggests the involvement of both hydrophobic and hydrogen bonds interactions. Seven hydrogen bonds were found involving Thr359 chain A with Gly3 chain B, Thr504 chain A with Asn22 chain B, Asn506 chain A with Met21 chain B, Asn506 chain A with Ser23 chain B, Lys610 chain A with Glu19 chain B, Tyr649 chain A with Asn22 chain B, Asn666 chain A with Ser23 chain B (Figure 2A).

The Mag-2/BamA model is shown in Figure 2B. The Gibbs free energy, ΔG, and the dissociation constant, Kd, of the protein-peptide complex were predicted using PRODIGY webserver obtaining the following values: ΔG = -14.6 Kcal/mol; Kd = 2.0E-11 M (at 25°C) suggesting the formation of a very stable protein-peptide complex. As the first and the last of 16th β-strands associate in closing the barrel to elicit BamA biological activity, the interaction with Mag-2 might have functional implications by contributing to stabilize the β-barrel domain in an open conformation and impairing the accommodation of OMPs within the barrel.

### 3.3 Recombinant production and purification of BamA_p5_

The hypothesis of a specific interaction occurring between Mag-2 and the BamA β-barrel domain suggested by molecular docking prompted us to confirm this putative binding on experimental basis using a recombinant form of BamA. The expression of the full-length BamA tagged with His-tail at the N-terminus resulted in a tiny amount of the protein showing a poor degree of purity. As the full-length BamA could not be produced in a satisfactory amount, as also reported in literature, we pointed out to a specific portion of the BamA protein that according to the docking predictions was involved in the interaction with Mag-2. The fragment encompassing the β-barrel and part of the fifth POTRA domain (residues 344-810) called BamAp5, was then produced in large amount and purified to homogeneity (Albrecht et al., 2014).

**FIGURE 3:**
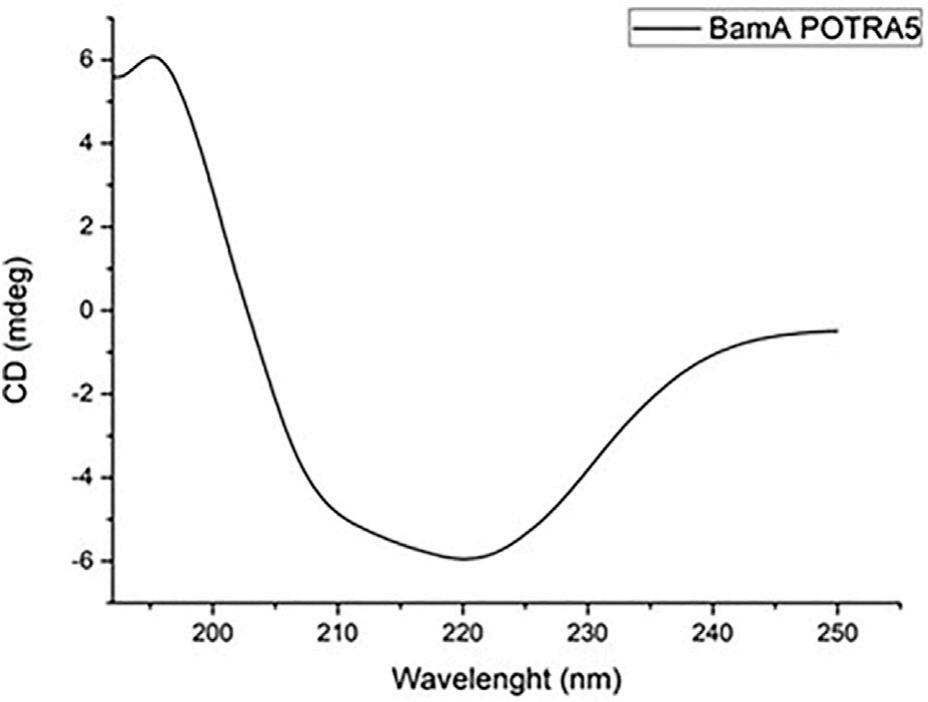
CD spectrum of 0.2 mg mL^−1^ BamA_p5_ protein in 20 mM Tris-HCl pH 8.4 buffer.

The purity of the protein was assessed by SDS-PAGE and its primary structure validated by peptide mapping with MALDI mass spectrometry. Circular dichroism analyses were also carried out to verify the correct folding of the protein. Figure 3 shows the corresponding CD spectrum displaying the expected ratio of α-helix and β-sheets secondary structures.

Molecular docking analysis of the complex between BamAp5 and Mag-2 was then performed suggesting again the formation of a stable complex with no relevant differences on the thermodynamics and kinetic parameters with the previous data with the entire BamA protein.

### 3.4 Binding experiments

**FIGURE 4:**
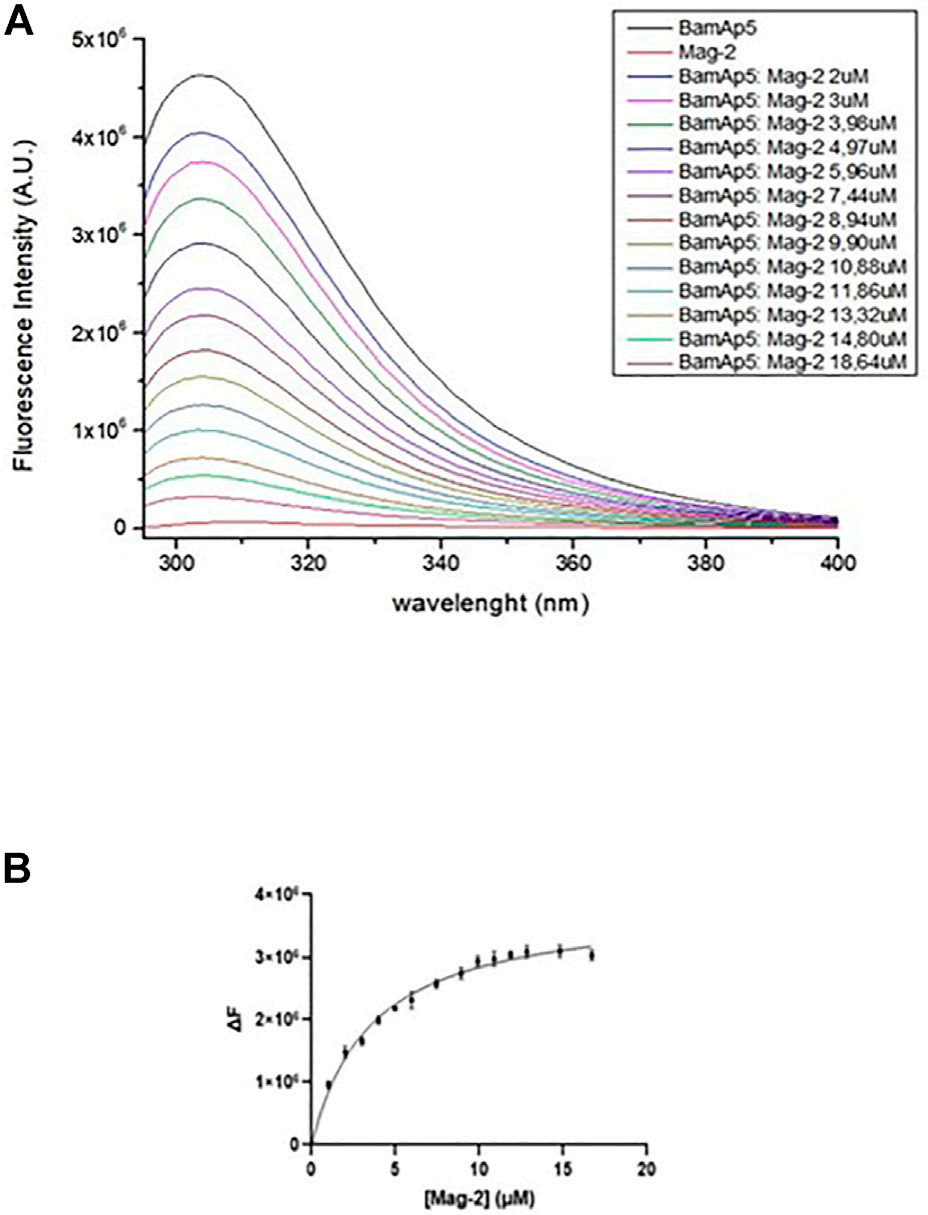
**(A)** Intrinsic fluorescence spectra (λexc = 280 nm) of 3.7 × 10^−6^ M BamA_p5_ by adding different concentration of Mag-2 every 5 min at 20°C. **(B)** Binding of Mag-2 to BamA_p5_ as determined by fluorescence experiments.

The binding of the Mag-2 peptide to BamA_P5_ protein was investigated by fluorescence assays in order to monitor changes in the tertiary structure of the protein. As BamAp5 has a large number of aromatic residues resulting in a high value of fluorescence intensity at 280 nm, the intrinsic fluorescence of BamA_P5_ was recorded upon incubation with increasing concentrations of Mag-2 avoiding the use of specific fluorescence tag/labels. The set of emission spectra of BamA_P5_ at various concentration of Mag-2 is shown in Figure 4. BamA_P5_ fluorescence decreased at increasing concentrations of the peptide confirming the occurrence of a peptide-protein interaction. Analysis of fluorescence data allowed us to calculate the dissociation constant of the complex, Kd value of 3.5 ± 0.1 nM confirming the formation of a stable protein-peptide complex and indicating a high affinity of Mag-2 for BamA_P5_.

### 3.5 DLS measurements

**FIGURE 5:**
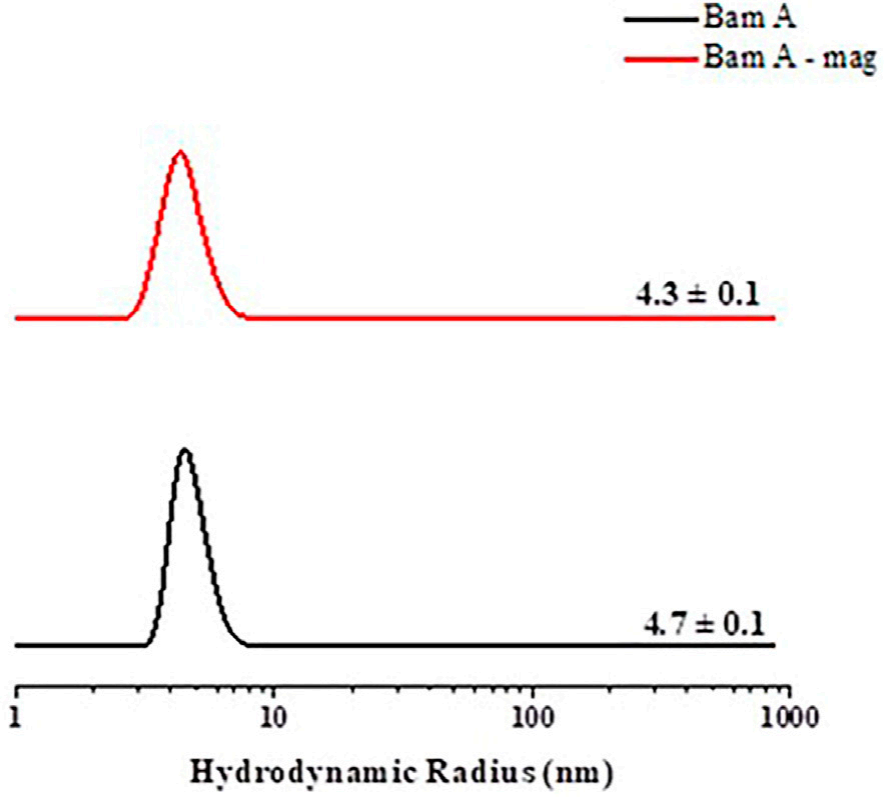
Hydrodynamic radius distribution of the aggregates in solution.

DLS measurements (Figure 5) were performed using 2 mg·ml-1 of Mag-2 and another sample with the same protein concentration but with a peptide concentration ten time higher than that of the protein. The measurement shows that only the protein monomer is present in both samples and is evident that the presence of peptide causes a reduction from 4.7 ± 0.1 to 4.3 ± 0.1 nm of the protein hydrodynamic radius. This well agree with the hypsocromic shift recorded with fluorescence spectroscopy and proves the protein-peptide complex formation.

### 3.6 Investigation of the functional properties of the Mag-2/BamA complex

The BAM complex plays a crucial role in the folding process of OMPs, including OmpA and OmpC that once correctly folded are released to distribute within the bacterium membrane. Interaction of Mag-2 with the BamA proteins should then prevent the proper binding of OMPs within the BamA β-barrel impairing their correct folding. Unfolded OMPs should then be degraded leading to a net decrease in the amount of these proteins. On these bases we were stimulated to investigate the effect of Mag-2 on the amount of OmpA protein produced by *E. coli* cells in the absence and in the presence of different sub-MIC concentrations of the peptide. Two experiments were developed using either Western Blot analyses or tandem mass spectrometry MRM investigations to evaluate the amount of OmpA following Mag-2 incubation.

#### 3.6.1 Western blot analyses

**FIGURE 6:**
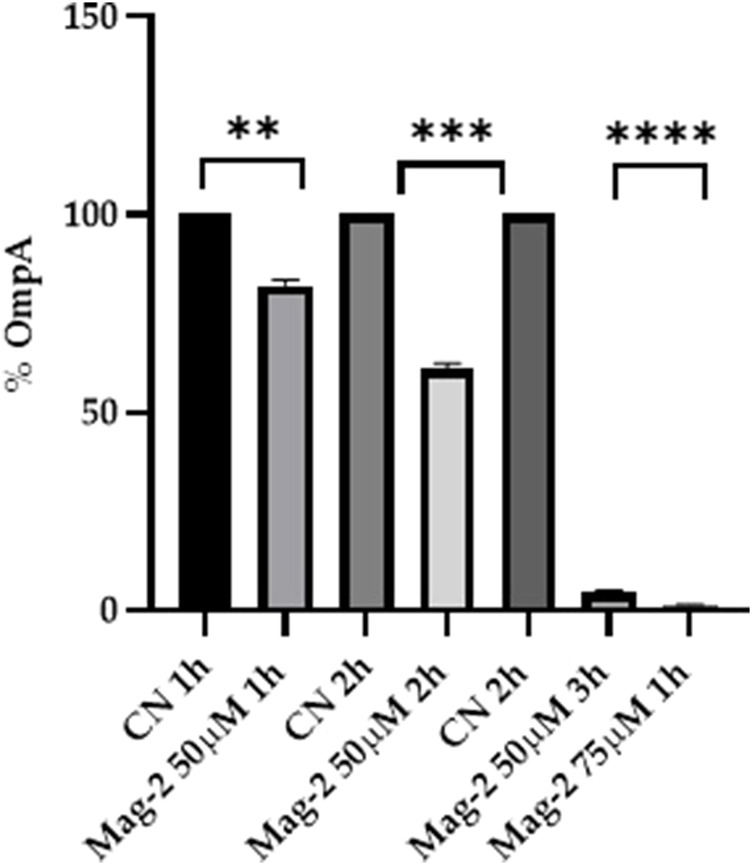
Densitometric analysis of the OmpA content carried out with Image Lab. The percentage of OmpA is shown on the *y* axis, the experimental conditions are shown on the *x* axis.

The *E. coli* cells were grown in the presence and in the absence of 50 µM Mag-2 for 1h, 2h and 3 h. The level of the OmpA protein was identified by Western Blot using a specific antiOmpA antibody. The amount of OmpA was then evaluated by densitometric analysis of the corresponding western blotted band. Figure 6 clearly shows that a gradual decrease in the amount of OmpA from 20 to 90% compared with untreated cells was observed as the time of incubation with the antimicrobial peptide increased. The same experiment was also carried out using a higher concentration of Mag-2 (75 µM) for as fixed time (1 h). The results are reported in Figure 6 showing a rapid and drastic reduction of the OmpA level, confirming previous data.

#### 3.6.2 Tandem mass spectrometry analysis in MRM scan mode

The amount of *E. coli* membrane proteins OmpA and OmpF were also evaluated by MRM tandem mass spectrometry following incubation with 50 µM Mag-2 for 1h, 2 h and 3h, the same conditions used for the Western Blot experiment. *E. coli* membrane proteins were extracted from the control and the sample, digested with trypsin and the resulting peptide mixtures were analysed in triplicate by using LC-MS/MS in MRM scan mode. A total of 18 peptides from both OmpA and OmpF target proteins were selected, and 140 transitions were monitored in a single analysis.

**FIGURE 7:**
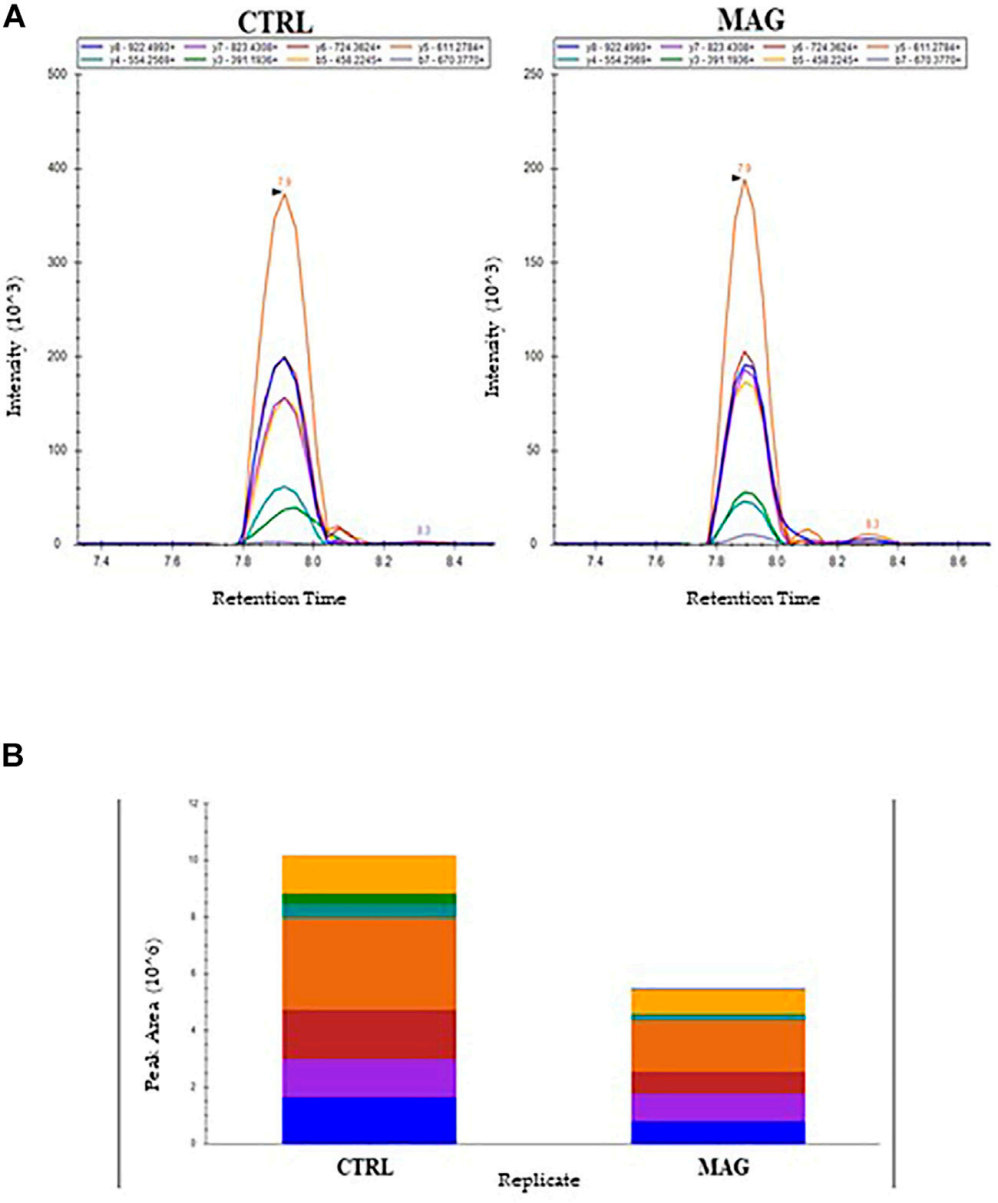
MRM Chromatogram for the 252-263 OmpA peptide (DGSVVVLGYTDR) in the control and Mag-2 treated *E. coli* samples. The seven best precursor ion-product ions transitions coeluted at 7.9 min Panel **(A)**. Quantitative comparison of peak areas is in Panel **(B)**

As an example, Figure 7A reported the perfect co-elution of all the monitored precursor ion-product ion transitions for the 252-263 OmpA peptide. Figure 7B shows the corresponding quantitative analysis of the recorded peak areas from which a decrease of the OmpA peptide in the sample treated with Mag-2 is observed compared to the control after 1 h of exposure.

**FIGURE 8:**
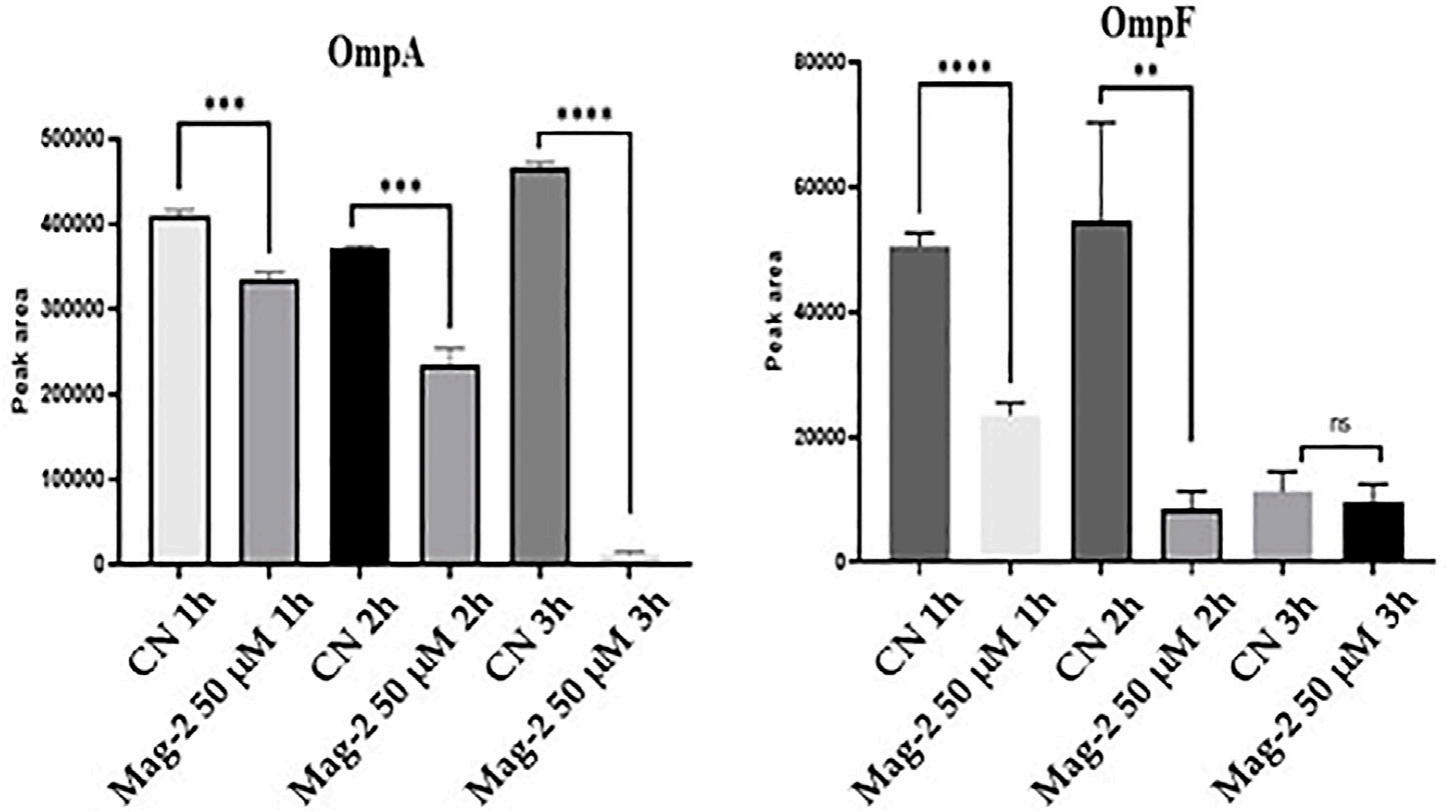
Quantitative measurements of OmpA and OmpF proteins at different times of incubation of *E. coli* cells with Mag-2 based on MRM/MS analyses. Statistical significance, *p*-value<0.05, was indicated as * in the figure.

The average peak areas from the different replicates were recorded for each peptide from OmpA and OmpF and their statistical relevance was confirmed by *t*-test (*p* < 0.05). The results of the quantitative evaluation of MRM/MS data are shown in Figure 8 where a clear decrease in the amount of both Omp and OmpF with increasing time of Mag-2 incubation was observed, confirming the results of the Western Blot experiments.

## 4 Discussion

Antimicrobial peptides are a unique and diverse group of molecules endowed with a broad spectrum of antibiotics properties. A number of biophysical studies were carried out to elucidate the AMPs molecular mechanisms demonstrating that the majority of AMPs are membrane-active molecules, killing bacteria by membrane disruption. This is also the case of Magainin-2, a well-known antimicrobial peptide isolated from the African clawed frog *Xenopus laevis*, whose mechanism of action has been extensively investigated. Mag-2 was suggested to interact with bacterial cell membranes forming pores in the lipid bilayers according to the so-called “toroidal” mechanism (Lee and Lee, 2015).

However, many recent studies showed that an increasing number of AMPs can also pass through the bacterial cell membrane and target intracellular processes essential for bacterial survival (Brogden, 2005). After entering the cell, AMPs can target nucleic acids and proteins, inhibit enzymatic activities affecting many crucial processes such as DNA and RNA replication, mRNA transcription, protein synthesis, cell cycle and energy metabolism (Le et al., 2017).

On this ground we were prompted to investigate whether Mag-2 might also have specific intracellular targets by designing appropriate functional proteomic experiments using a biotinylated version of the peptide as a bait to fish its putative interactors out from *E. coli* membrane. Proteomic results suggested that Mag-2 might interact with several proteins belonging to multicomponent membrane complexes involved in different functions. Among these putative Mag-2 partners, we identified many components of the β-barrel assembly machinery (BAM) complex, namely BamA, BamB, BamC and BamD. In Gram-negative bacteria, the essential BAM participates in the outer membrane proteins (OMPs) assembly, although it is currently unclear how the BAM complex functions in OMP folding and insertion (Hagan et al., 2015). However, according to the pore-folding model, the β-barrel of BamA offers its pore for insertion of the nascent OMP into the membrane, and the POTRA (polypeptide transport-associated) domains or accessory components act to thread the OMP into the pore. The insertase BamA is the central protein of the complex forming interaction with four lipoprotein partners BamBCDE in *E. coli*. However, it is thought that after delivery of unfolded OMPs to a large ß-barrel cavity within BamA structure, the protein undergoes a lateral opening of the barrel through which the folded OMPs travel to their final destination in the OM bilayer (Schiffrin et al., 2017). Disulphide crosslinks that prevent lateral opening result in a loss of BamA function, providing strong evidence that lateral opening is required for BamA function (Noinaj et al., 2014).

We were then stimulated to investigate whether the interaction of Mag-2 with BamA might inhibit BamA function and impairing OMPs folding. Investigation of this interaction *in silico* by molecular docking simulation suggested the formation of a very stable BamA/Mag-2 complex with a dissociation constant in the low nanomolar range. Fluorescent binding experiments performed using a recombinant version of BamA in the presence of different concentrations of Mag-2 confirmed molecular docking predictions showing the formation of a stable BamA/Mag-2 complex with a low nanomolar value of Kd indicating a high affinity of the peptide for BamA.

Finally, we tested whether the BamA-Mag-2 interaction might affect the biological functions of the BAM complex using two different and complementary approaches. In both cases a quantitative evaluation of the total amount of specific OMPs in *E. coli* cells was performed in the presence and in the absence of Mag-2. First, the amount of OmpA produced by *E. coli* cells following incubation with a subMIC concentration of Mag-2 was estimated by Western Blot using a specific antiOmpA antibody. The presence of the peptide clearly affected the production of OmpA as a gradual decrease in the amount of the protein compared with untreated cells could easily be observed. These data were confirmed by a more sophisticated experiment using tandem mass spectrometry in MRM scan mode. Several specific peptides from both OmpA and OmpF were selected and their mass transitions from the molecular ion to individual fragment ions were predicted using the Skyline software. The MRM analyses confirmed the occurrence of the selected peptides by identification of the corresponding predicted mass transitions. Quantitative evaluation of MRM mass spectrometry data was in prefect agreement with previous Western Blot results indicating a gradual decrease in the production of OMPs when *E. coli* cells were previously incubated with Mag-2.

Overall, the data reported in this paper led to an accurate hypothesis on the mechanism of action of Mag-2. Besides its activity on the bacterial cell membrane, the antimicrobial peptide crosses the outer membrane and specifically binds BamA within the large cavity of the β–barrel structure. The peptide makes contact with several key residues involved in the functional mechanism of the protein impairing the proper folding and allocation of the outer membrane proteins according to either of two possible effects. The interaction of Mag-2 with BamA might physically prevent the unfolded OMPs to enter the β–barrel cavity to correctly complete the folding process. Alternatively, binding of the peptide might prevent the conformational changes needed to disrupt the unstable junction between ß-strands 1 and 16 impairing the opening of the lateral gate through which the folded OMPs are released from the BAM complex enroute to their final location in the OM bilayer.

Mag-2 showed a low but significant toxicity against human cells and therefore cannot be considered as an effective alternative to common antibiotics. Nevertheless, identification of BamA as a specific Mag-2 intracellular target pointed out to this protein as a possible good target for the rational design of new antibiotics since this protein is responsible for a crucial biological event of bacterial life and is absent in humans.

## Data Availability

The mass spectrometry proteomics data have been deposited to the ProteomeXchange Consortium via the PRIDE partner repository (Perez-Riverol et al., 2022) with the dataset identifier PXD037418.
