# Supplementary figures and images for: The antimicrobial peptide Magainin-2 interacts with BamA impairing folding of E. coli membrane proteins
Source: Front Chem. 2022 Oct 17;10:1013788. doi: 10.3389/fchem.2022.1013788 (PMC9620421; doi:10.3389/fchem.2022.1013788)

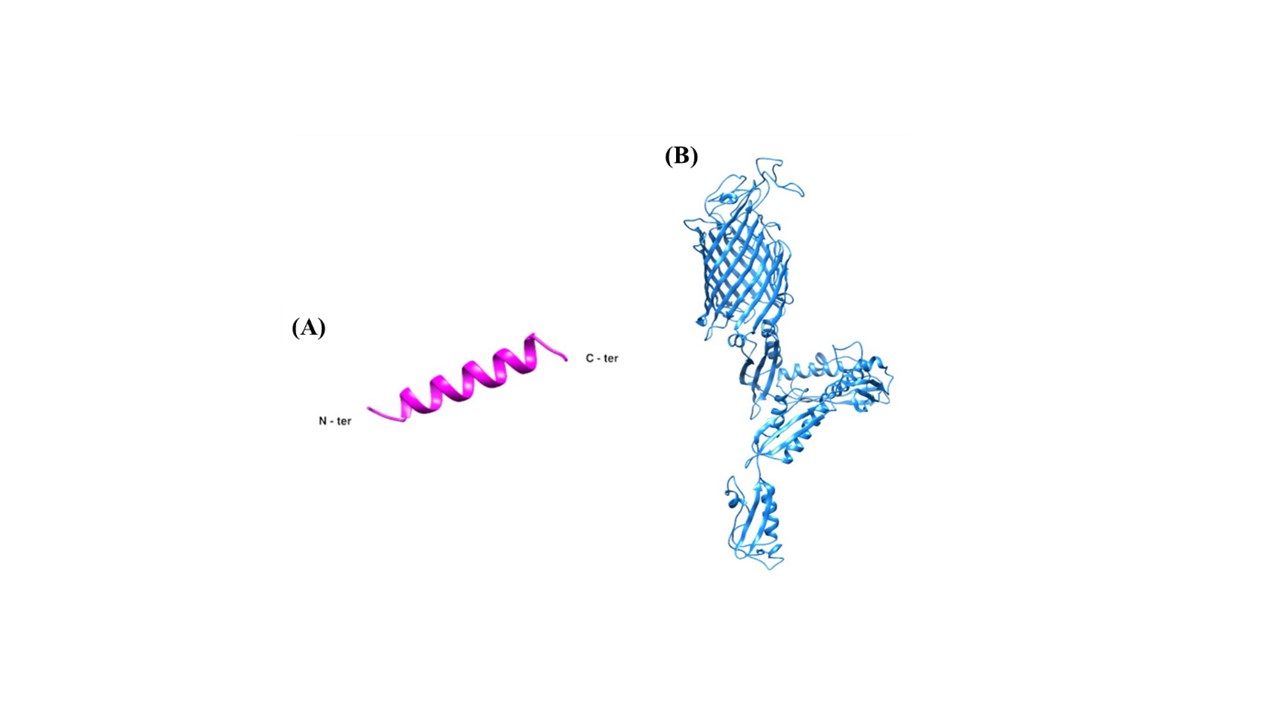

Supplement: Supplementary file 1 [file Image1.JPEG]
